# Using a citizen science approach to assess nanoplastics pollution in remote high-altitude glaciers

**DOI:** 10.1038/s41598-024-84210-9

**Published:** 2025-01-13

**Authors:** Leonie Jurkschat, Alasdair J. Gill, Robin Milner, Rupert Holzinger, Nikolaos Evangeliou, Sabine Eckhardt, Dušan Materić

**Affiliations:** 1 Faculty of Chemistry and Mineralogy, Leipzig University, 04103 Leipzig, Germany; 2 Department of Environmental Analytical Chemistry, Helmholtz Centre for Environmental Research (UFZ), 04318 Leipzig, Germany; 3 Institute for Marine and Atmospheric Research (IMAU), Utrecht University, Utrecht, 3584 The Netherlands; 4 Department of Atmospheric and Climate Research (ATMOS), NILU, Kjeller, 2007 Norway; 5 High Level Route, https://www.high-level-route.com

**Keywords:** Microplastics, Nanoplastics, Thermal desorption-proton transfer reaction-mass spectrometry (TD-PTR-MS), Atmospheric transport, Citizen science, Environmental sciences, Environmental chemistry

## Abstract

Nanoplastics are suspected to pollute every environment on Earth, including very remote areas reached via atmospheric transport. We approached the challenge of measuring environmental nanoplastics by combining high-sensitivity TD-PTR-MS (thermal desorption-proton transfer reaction-mass spectrometry) with trained mountaineers sampling high-altitude glaciers (“citizen science”). Particles < 1 μm were analysed for common polymers (polyethylene, polyethylene terephthalate, polypropylene, polyvinyl chloride, polystyrene and tire wear particles), revealing nanoplastic concentrations ranging 2–80 ng mL^− 1^ at five of 14 sites. The dominant polymer types found in this study were tire wear, polystyrene and polyethylene particles (41%, 28% and 12%, respectively). Lagrangian dispersion modelling was used to reconstruct possible sources of micro- and nanoplastic emissions for those observations, which appear to lie largely to the west of the Alps. France, Spain and Switzerland have the highest contributions to the modelled emissions. The citizen science approach was found to be feasible providing strict quality control measures are in place, and is an effective way to be able to collect data from remote and inaccessible regions across the world.

**Supplementary Information:**

The online version contains supplementary material available at 10.1038/s41598-024-84210-9.

## Introduction

Micro- and nanoplastics pollution is a well-known problem and extensive research has tried to quantify the amount of plastic released into the environment as well as investigate its negative effects on living organisms^1–3^. Although there is not yet a single clear definition for nanoplastics, they are are generally defined by a size of < 1 μm in at least one dimension^3^. The main source of nanoplastics in the environment is the degradation of macro- and microplastics by both abiotic and biotic processes (e.g. photo-, mechanical, oxidative, hydrolytic and enzymatic degradation^2,3^). There is ample evidence of microplastics in remote and high-altitude environments across the world, including in snow and glaciers in the Alps, Andes, Himalayas, the Tibetan Plateau and on Iceland^4–7^. As microplastics have been found almost everywhere, we must logically expect nanoplastics almost everywhere as a result of degradation. In addition, atmospheric transport is already known to bring microplastics to remote regions^7–9^; this is likely to be even more significant for nanoplastics due to their smaller size and weight.

However, analysis of nanoplastics is challenging – mass spectrometry-based methods generally require laborious preconcentration steps and spectroscopic methods are only partially applicable at this scale due to the diffraction limit of light. In recent years, thermal desorption-proton transfer reaction-mass spectrometry (TD-PTR-MS) has emerged as a highly sensitive method able to detect nanoplastics in complex environmental samples, with the limit of detection (LOD) for polystyrene (PS) estimated at < 1 ng^10^. The increased sensitivity eliminates the need for preconcentration, reducing the sample volume as well as the risk of contamination.

These improvements led us to test a novel “citizen science” approach by collaborating with the High Level Route (HLR) mountaineering expedition^11^. Covering remote Alpine glaciers, specially trained mountaineers systematically sampled otherwise inaccessible areas, giving us the opportunity to investigate the nanoplastics situation at high altitude. Assuming direct pollution sources such as traffic and litter can be excluded due to the remoteness, this gives us an idea of the amount and composition of nanoplastics deposited on glaciers through airborne transport.

## Methods and materials

### Sampling and analysis by TD-PTR-MS

**Fig. 1:**
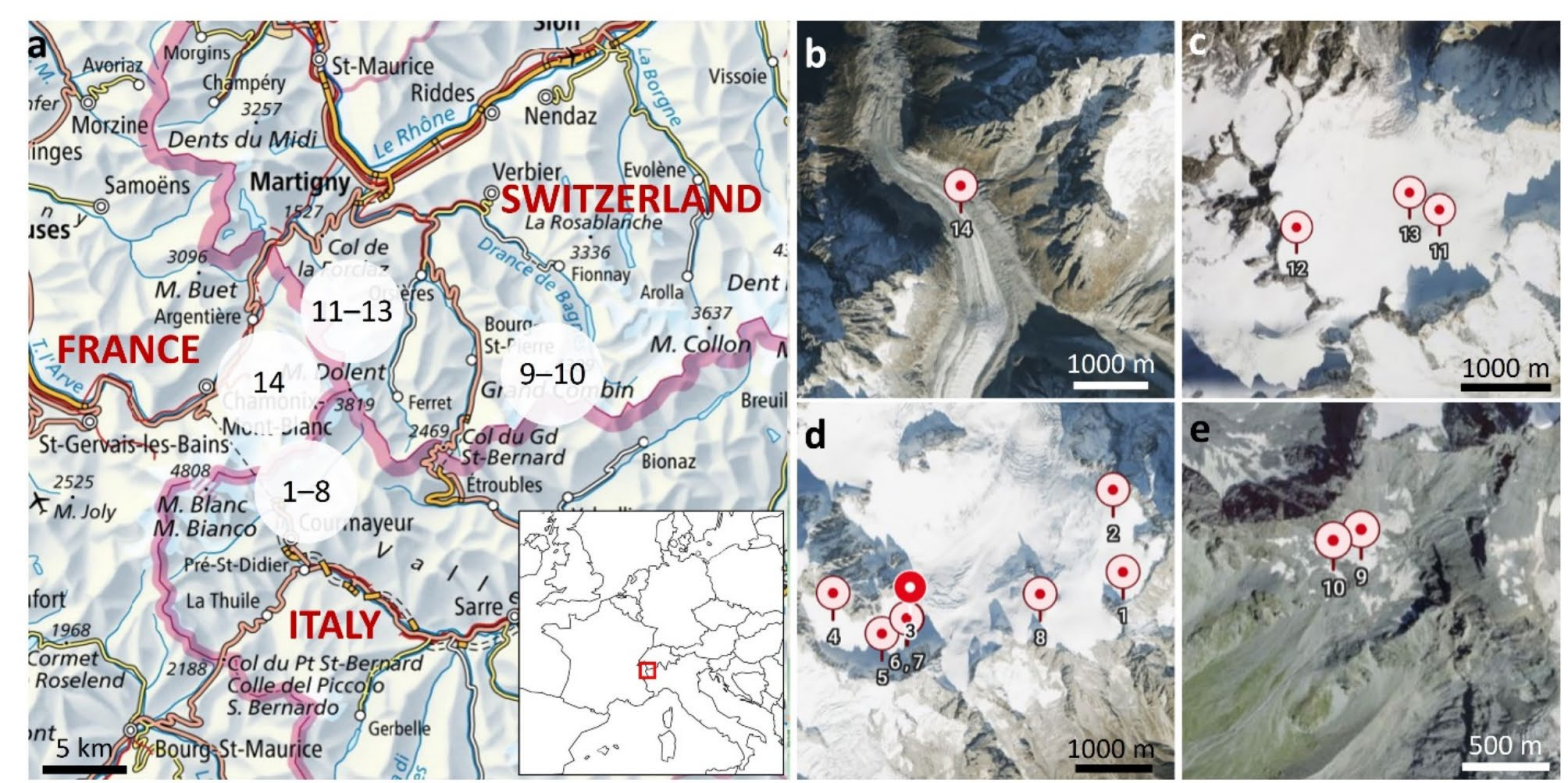
Map of the sites sampled in the Alps during the 2021 reconnaissance expedition. (a) Location within Europe. **(b)–(e)** Satellite images showing exact location of sample sites in the terrain. Created using Bing maps, URL links map.geo.admin.ch and d-maps.com^12–14^.

Samples of ca. 100 mL surface snow were taken during the HLR reconnaissance expedition in August 2021. A map of the sampling sites is shown in Fig. 1 (see also Supplementary Information Fig. S1 and S2). A standard operating procedure (SOP) for the sample-taking was developed together with the mountaineers. A pre-cleaned sampling kit was provided, with all vials baked at 250 °C overnight and closed with new, polytetrafluoroethylene (PTFE)-coated lids. To minimise risk of plastics contamination, the sampler wore new gloves for sampling, wore the same outer clothes and stored vials in a cotton bag in the rucksack (for the complete SOP see Supplementary Information). Thirteen sites were sampled (all above 3100 m except site 14) and one site was sampled twice within 10 m as a control (hereafter treated as 14 sites). Samples were taken in triplicate (apart from site 14, only duplicate due to the technical challenge of sampling within a crevasse) and a field blank was taken at each site, where high-pressure liquid chromatography (HPLC)-grade water was poured into the clean vial on site and further handled in the same way as the snow. The samples were received as melted snow and had been stored at ambient temperatures away from light since collection.

The sample preparation followed our standard procedure^10,15–18^. A volume of 5 mL of each sample and filed blank were filtered through a 1 μm-mesh disposable PTFE syringe filter (Machery-Nagel) using a polypropylene (PP) Luer-slip syringe (Labsolute). The field blanks were processed in the same manner as the snow samples to ensure the quality of the results. In addition, two samples and two field blanks were each spiked with 300 ng of PS (0.5 μm nanoparticles, analytical standard, Sigma Aldrich). The filtered samples were stored in pre-baked 10 mL glass vials closed with PTFE discs (in house production) under aluminium screw caps (VWR). From each filtered sample/field blank, 1 mL was transferred into a new vial, closed with a perforated PTFE disc and vacuum-dried in a desiccator until the water had completely evaporated^19^. Nanoplastics analysis was performed on a PTR8000 PTR-MS (IONICON Analytik) as described in detail before^10,16,17,20,21^. The thermal desorption (TD) program was 35 °C for 30 s, ramp with 40 °C min^− 1^ to 360 °C, plateau at 360 °C for 3 min. Two system blanks (clean, baked vials) were measured at the beginning and one at the end of each day of measurement.

### Data processing

**Fig. 2:**
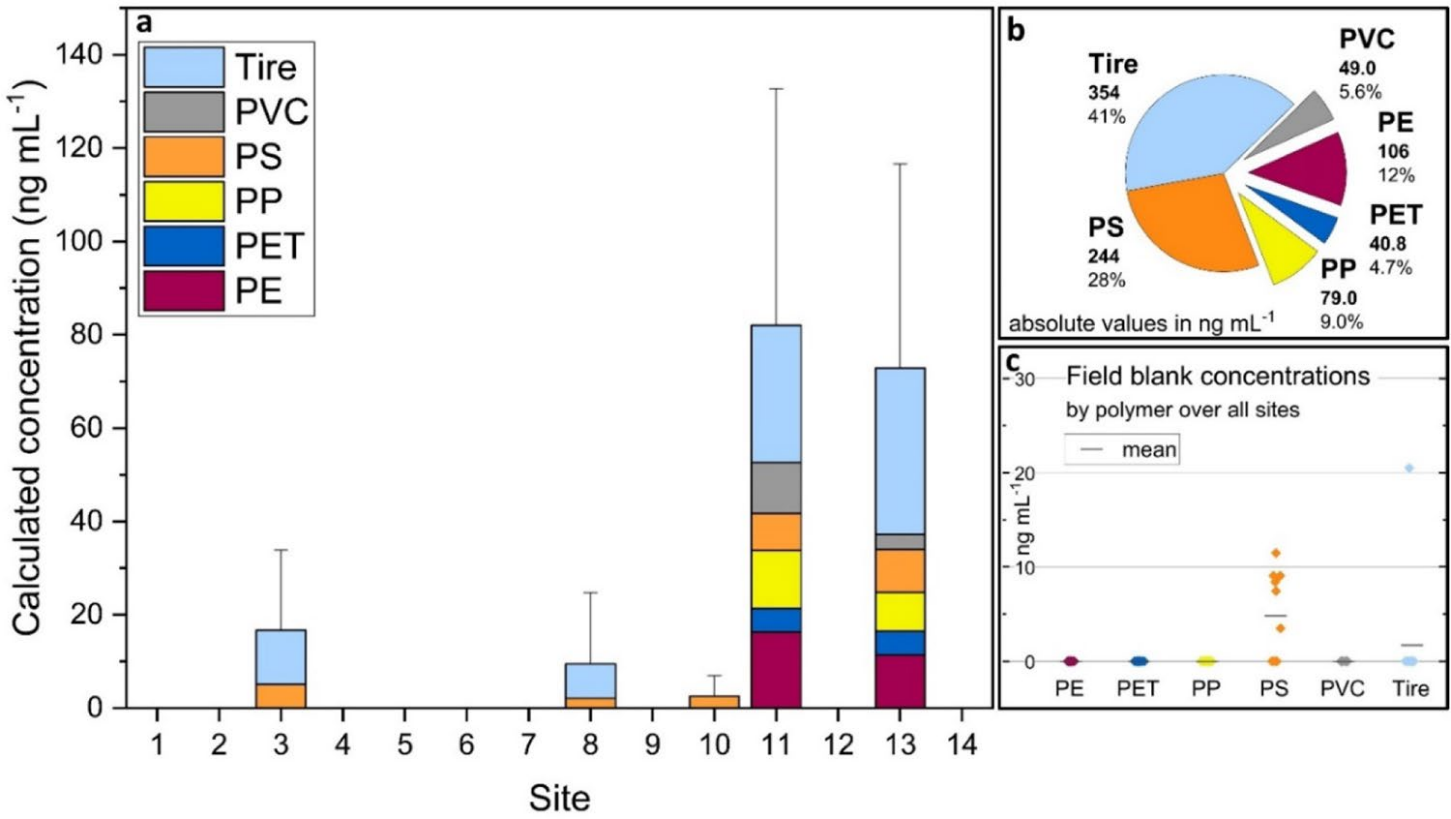
Calculated mass concentration of nanoplastics detected at Alpine sites during the 2021 reconnaissance expedition. (**a**) Mass concentration of tire wear particles, polyvinylchloride (PVC), polystyrene (PS), polypropylene (PP), polyethylene terephthalate (PET) and polyethylene (PE) at each site, mean field blank concentrations subtracted. Mass concentrations here represent the lower threshold values and are not corrected for recovery/ionisation efficiency (e.g. recovery of PS is ca. 25%, see Supplementary Information, Tab. S2). Error bars show quadrature sum of standard deviations of each polymer. **(b)** Total mass concentration of each polymer over all sites. **(c)** Nanoplastics detected in field blanks. For all calculated values of individual samples and field blanks see Supplementary Information, Table S1.

Data extraction was performed as described in previous work^16,17^. In short, mass spectra are integrated and concentration in ppb (parts per billion) calculated using the PTRwid tool^22^ (version PTRwidv003may182022) from the ion count, instrument parameters and known kinetics of the proton transfer reaction. A detailed description of PTR-MS method can be found in the literature^23–26^. Polymers were identified and quantified using TD-PTR-MS method and reference spectra and calibrations as described previously^10,16^. The polymer mass spectra library (including the tire wear particles) with all organic ions (m/z and intensities) are available in our previous work^10,20^ (see also Data and Code availability section). The mass spectra were integrated over 7 min from when the TD unit reached 200 °C and the 40 highest-intensity ions were used for fingerprinting. The mean blank concentration for each ion was calculated using the system blanks (*n* = 17) and subtracted from the measured concentration before fingerprinting; values below the LOD (3σ, threefold standard deviation of system blanks) were not considered for analysis. The final calculated nanoplastic loads were corrected by subtracting the mean field blank concentration of each polymer (see Fig. 2).

### Quality control, quality assurance and data limitation

Due to the near-ubiquitous presence of plastics, strict measures were taken to minimise contamination during sampling, transport, storage and handling in the laboratory, to ensure the data are as accurate as possible^27^. These also needed to be practical and feasible during a mountaineering expedition, and are described in Sect. 2.1 and the Supplementary Information. Samples were wrapped in aluminium foil and stored at ambient temperatures away from light until analysis. Vials were only opened in a laminar flow cabinet wearing new nitrile gloves and exposure to laboratory air was minimised.

Field blanks were taken to check for possible contamination. Polyethylene (PE), polyethylene terephthalate (PET), PP and polyvinyl chloride (PVC) were not detected in any field blanks, tire wear particles in one and PS in seven (see Fig. 2c). This shows that for the majority, contamination with the fingerprinted polymers was negligible. Despite some laboratory materials being of plastic (e.g. PP pipette tips, PP syringe), we detected no increase in these polymers in our blanks, which is in line with our previous work^10,17^ and recently confirmed by others, where new plastic consumables outperformed glass consumables^28^. As tire wear particles were only detected in one blank, we assume an incidental point contamination, however, the detection of PS in several indicates a possible systematic contamination. The source of this could be PS particles in the air either at the sampling site or in the laboratory; expedition equipment is unlikely as PS is not typical for clothing and was not used for transport. Additional sets of blanks, e.g. “carry blanks” exposed to the same expedition and transport equipment without being opened, as well as laboratory process blanks, should in future be used to identify the source.

System blanks allowed for an instrument background correction and served as a filter to remove signals below the estimated LOD (3σ limit) as described previously^21^. The LOD was calculated as 2.4 ng mL^− 1^ for PS and 0.7–1.2 ng for PET using calibration with standard materials (details see Supplementary Information, Fig. S3 and S4, Tab. S3), which differs slightly from previous work, where 0.34 and 0.12 ng mL^− 1^ was calculated for PS and 2.6 and 7.7 ng mL^− 1^ for PET^10,18,20^. However, this still represents an extremely sensitive analysis in the ng range without any preconcentration steps required. Analytical standards of nanoplastics of other polymers are not yet commercially available and so for these, an LOD could not be experimentally estimated.

To estimate recovery/ionisation efficiency, random samples were spiked with a known amount of PS nanoparticles (0.5 μm, analytical standard, Sigma Aldrich). We measured an average of 73.9 ng out of spiked 300 ng (details see Supplementary Information, Tab. S2). This indicates a recovery/ionisation efficiency of 25%, which compares to previous work (15%^10^, 20%^20^ ad 31%^18^). Th accuracy of quantification via PTR-MS has been found to be ± 30%^24^, and an verall uncertainty of ca. 60% has been discssed previously^16^. All values are given without correction for recovery and should be considered semi-quantitative, conservative estimates, thus a minimum threshold of real values. The successful fingerprint of spiked samples of z-score > 3^10^ indicates minimal or no matrix effect on our analysis.

Sampling in triplicate and including a control sample allowed us to assess the quality and limitations of the sampling process. The data show relatively large variation between triplicates, which we believe reflect the challenging nature of the analysis and environment rather than inexpert sampling by mountaineers. With discrete particles of unknown size, there is an element of chance in how many of these in a sampled volume are analysed due to aliquots being taken. However, the reproducibility between control sites 6 and 7, taken within 10 m of each other, was good (see Fig. 2a). It is unclear how uniform nanoplastics are likely to be distributed across glacial snow and more replicates per site or analysing the total volume of melted snow would provide a more accurate picture.

It is important to note here that non-detection can mean several things. Firstly, no plastic was present in the snow or the quantities were below the detection limit; secondly, plastic was present but the particles were too large to pass through the 1 μm filter or were not in one of the 1 mL aliquots analysed; thirdly, other less common plastic was present for which a fingerprint has not yet been added to the library. Furthermore, TD-PTR-MS cannot give information on the size distribution, shape, particle number or presence of additives. For a more complete picture, other techniques such as Raman spectroscopy, scanning electron microscopy, nanoparticle tracking analysis or pyrolysis-GC-MS could be helpful. However, at the time they were not available, considering the small sample quantity and required detection limit.

### Lagrangian dispersion modelling

To track the long-range transport of the deposited atmospheric micro- and nanoplastics, the Lagrangian particle dispersion model FLEXPART version 11^29^ was used driven with hourly ERA5 assimilated meteorological analyses^30^ of 137 vertical levels, a temporal resolution of 3 h and a horizontal resolution of 0.5° x 0.5°. The footprint emission sensitivities (FES) were calculated in backwards time mode, using a feature that reconstructs wet and dry deposition at the receptor for backward simulations^31^.

Wet deposition of microplastics was reconstructed after releasing computational particles at each receptor (sampling site) at altitudes of 0–20 km above sea level during precipitation events. Scavenging was calculated with different removal rates for cloud condensation nuclei within clouds and rainout below clouds. For dry deposition, particles were released at 0–30 m at the same receptor, as this shallow layer is equal to the height of the layer in which, in forward mode, particles are subject to dry deposition. All released particles represent a unity deposition amount, which was converted immediately (i.e. upon release of a particle) to atmospheric concentrations using the deposition intensity as characterised by either the dry deposition velocity or wet scavenging rate (in-cloud and below-cloud scavenging^31,32^). The termination time of the particle release was the time at which the snow sample was collected, whereas the beginning time was set as the time when the ERA5 precipitation at the sampling site, accumulated backward in time, was equal to the water equivalent of the snow sample up to the specified sampling depth. This gives the sensitivities between emissions and deposition amounts using 50-day backward tracking (previous calculations have shown that given the atmospheric lifetime of nanoplastics, 50 days should be able to capture > 98% of te sources).

In the present study, a beta version suitable to model micro- and nanoplastics was used, which takes into consideration shape corrections during gravitational settling in order to account for shapes other than spheres^33^. However, this is less important for nanoplastic sizes. We have assumed that the modelled microplastics are fragments that, in turn, we define as cylinders with a diameter of base of 1 μm (to account for nanoplastics size) and 10 μm (to account for microplastics size) and an aspect ratio of 30 (that defines the length of the cylinder). The reason why fragments were chosen as the prevailing shape is that they represent most of the shapes that are usually found in observations (fibres and fragments)^34,35^. They also have an atmospheric lifetime that is larger than that of spheres and smaller than that of fibres^33^; thus more representative of the micro- and nanoplastics.

The model output consists of a spatially gridded sensitivity to the respective emissions for the microplastic deposition (wet and dry) for each receptor point^31^. Deposition rates of microplastics (in kg m^− 2^ day^− 1^) can be computed by multiplying the emission sensitivities (in m) with gridded emissions (in kg m^− 2^ day^− 1^), divided by the lowest model layer (100 m). Multiplying by the duration of the accumulation of snow, as defined by the water equivalent volume of each sample, gives the modelled concentration. In the present study, we used global emissions for microplastics and microfibres from previous work^36^.

## Results and discussion

### Nanoplastics detected on glaciers

Presence of nanoplastics has been detected for five out of 14 sites, mass concentration ranging 2–80 ng mL^− 1^, with tire wear, PS and PE particles making up the majority by mass (41%, 28% and 12%, respectively), see Fig. 2. As these are very remote sites and almost never frequented by people, finding nanoplastics here is significant and points to airborne transport. The contribution of different polymers differs slightly from previous results in the Alps, where PET and PP dominated in melted snow (5–23 and 29.5 ng mL^− 1^, respectively^10,17^) and PET, PP and PE in atmospheric particulates (average concentration 21 ng m^− 3^^18^). In a remote monitoring station in the central Pyrenees, France (1425 m altitude), an average deposition rate of 50 ng m^− 2^ day^− 1^ was found for the < 0.45 μm fraction. The dominant polymer varied between months^37^. Looking at non high-altidude glacial/ice environments, in a Greenland firn core, PE was also the most abundant, followed by tire wear and PET (6.5, 3.2 and 2.7 ng mL^− 1^, respectively), whereas in an Antartica sea ice core, it was PE and PP (38 and 20.7 ng mL^− 1^ at top of core, respectively^16^).

Although different nanoplastic polymers have been found to be most abundant, which may be due to different transport mechanisms and distance from sources, the same few, namely PE, PP, PET and tire wear particles dominate across the Earth. The absolute mass concentrations in aqueous samples are all in the tens of ng mL^− 1^, which agrees well with the results from this work. Of the sites where nanoplastics were detected, all had a positive PS fingerprint. At sites 3 and 8 tire wear particles were found in addition to PS and at sites 11 and 13 all polymers in the library were found. Sites 11 and 13 also show a much higher total concentration of plastics than the other sites. One factor that may play a role here is the topography (see Fig. 1c and S2, Supplementary Information). Sites 11 and 13 are situated in the centre of a hollow, which may favour wet and dry deposition, whereas site 12 is closer to the ridge, which possibly shields it from prevailing westerly winds carrying nanoplastics to the Alps.

Judging from the quality of our samples and blanks, the citizen science approach proves to be a promising method for gathering data in remote and untraveled areas that are difficult for research teams to access without specialized skills, such as mountaineering or polar navigation. At the same time, it raises awareness about plastic pollution among a broader audience, fostering public engagement and education on environmental issues. When carefully supervised and conducted according to standardized protocols, crowd-sourced sample collection enables researchers to gather data from multiple sites within a short timeframe. This approach not only accelerates data collection but also reduces costs and expands the geographical reach of environmental studies, ultimately contributing to a more comprehensive understanding of global plastic pollution.

### Potential sources of deposited micro- and nanoplastics

**Fig. 3:**
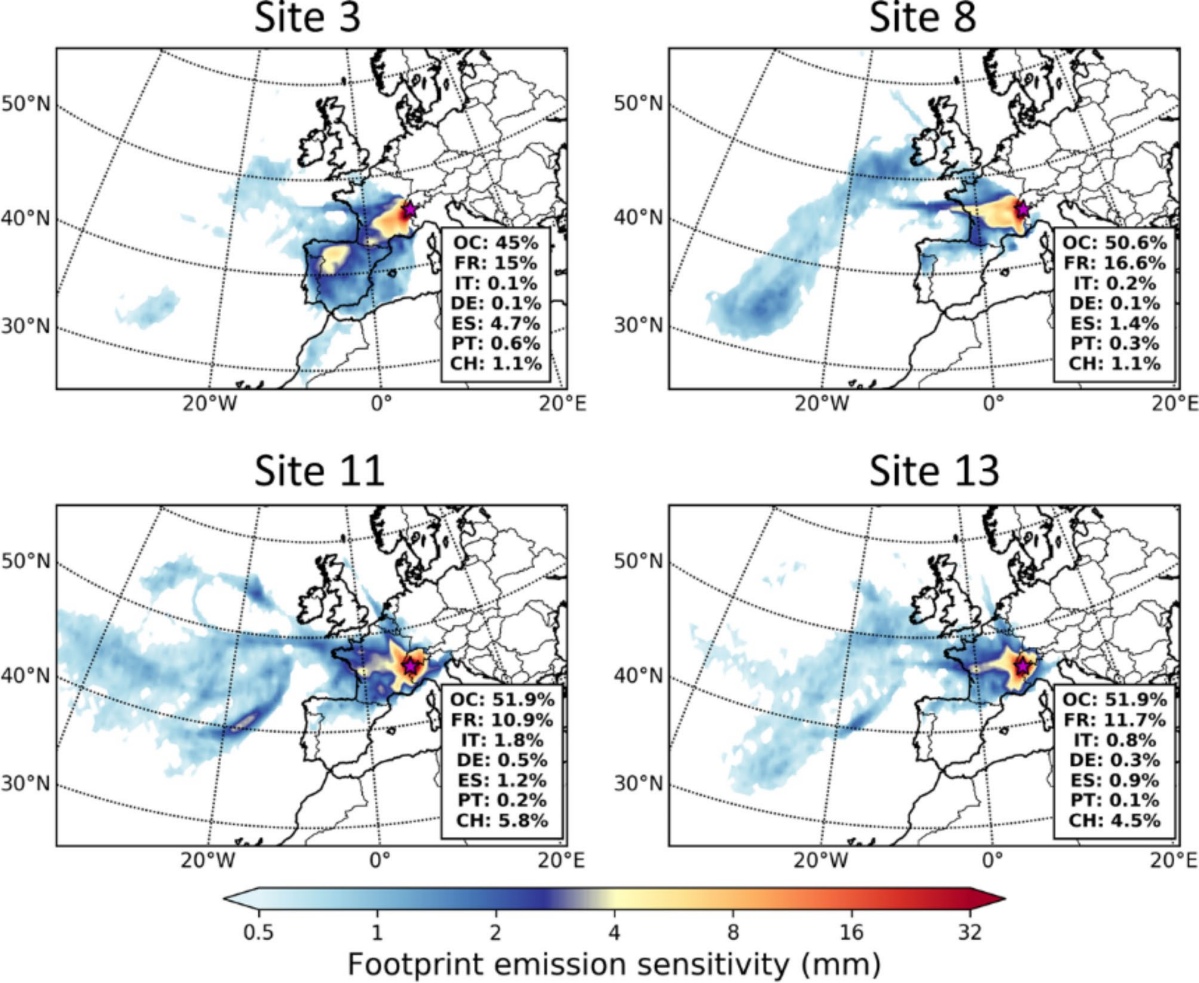
Lagrangian dispersion modelling: footprint emission sensitivity (FES) for the sites with largest observed deposition rates detected during the 2021 reconnaissance expedition (sites 3, 8, 11 and 13). FES represents emission probability. Nanoplastics most likely originated from the west, travelling via airborne transport until they were removed from the atmosphere and deposited at the sites in the Alps. For all sites, the ocean represents the highest contribution to FES, followed by continental contribution from France; contribution from other countries shown in inset (OC = ocean, FR = France, IT = Italy, DE = Germany, ES = Spain, PT = Portugal, CH = Switzerland).

The respective footprint emission sensitivities for each receptor and site are shown in https://atmo-access.nilu.no/MPS_HLR1.py, while the FES for the largest observed deposition rates (sites 3, 8, 11 and 13) are depicted in Fig. 3 in the nanoplastic size (< 1 μm). FES is a measure for emission probability, i.e. the higher the value in a grid cell, the larger the probability that the particles originated from there.

In most of the cases, the deposited nanoplastics originate from the west, getting substantial contribution from the Atlantic Ocean (> 45%) and France (> 10%). The FES for sites 3, 8, 11 and 13 in Fig. 3 is a result of two processes usually occurring when aerosols are suspended, namely dry and wet deposition.

Dry deposition is greatly influenced by the settling velocity that depends on the size and density of the shapes to be modelled (here non-spherical shapes were used). For nanoplastics, due to their small size, the proportion of dry deposition is generally very low. Wet deposition is a result of in-cloud and below-cloud scavenging, which depends on respective scavenging coefficients for cloud condensation nuclei and ice nuclei.

**Fig. 4:**
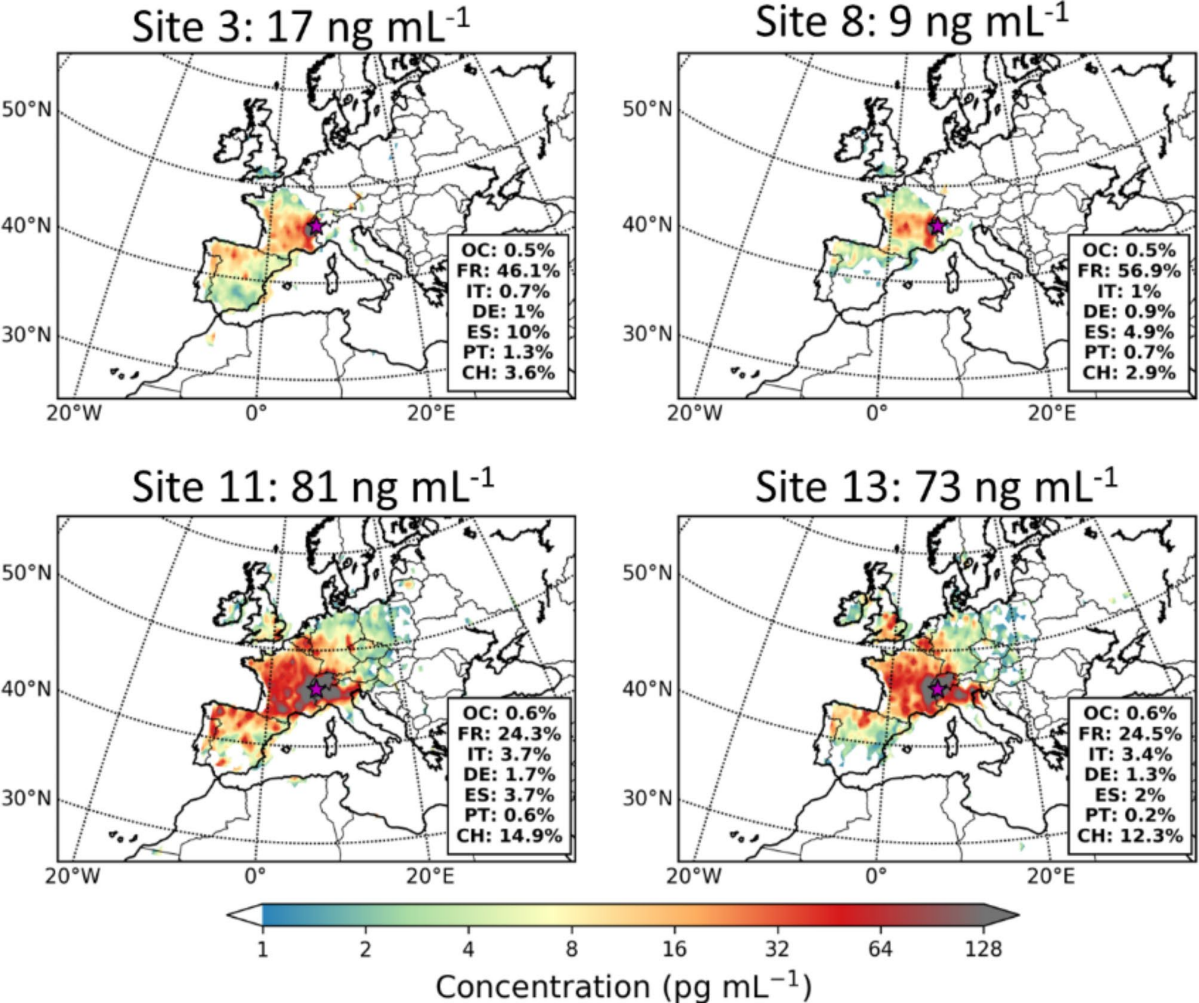
Lagrangian dispersion modelling: contribution to nanoplastics deposition, for the sites with largest observed deposition rates detected during the 2021 reconnaissance expedition (sites 3, 8, 11 and 13), using emissions from Evangeliou et al.^36^. The maps show the spatial distribution of the contribution to each site as gridded values, where the sum of all grid cells equals the modelled total (dry and wet) deposition at the site, which can be directly compared to the observation (shown at top for each site). France, Spain and Switzerland appear to be the largest contributors to the deposited microplastics sampled in the Alps.

Considering that plastics are hydrophobic polymers, one would expect that coefficients characterizing lower scavenging than many conventional aerosol species are to be used. However, recent studies suggest that a transition from hydrophobic to hydrophilic occurs due to atmospheric ageing^38,39^.

Since the analytical technique used cannot give any information about the shapes of the measured nanoplastics, we performed a sensitivity study to calculate the associated uncertainty with respect of the different shapes used in the model. We considered spherical particles of diameters the same as the measured ones (1 and 10 μm), fragments as those defined in Sect. 2.4 and fibres with 1 and 10 μm diameter base and an aspect ratio of 100. The uncertainty of transport was calculated as the standard deviation of the resulting FES. The results are shown in the Supplementary Information, Fig. S5. We see that the average uncertainty is under 5% compared to the FES in Fig. 3. The largest uncertainty is obtained far from the receptors; this is more or less expected, as fibres are dispersed longer than fragments and, in turn, fragments are distributed longer than spheres. In relation to this, Tatsii et al.^33^ reported that settling velocities of fibres are reduced by up to 76% cmpared to those of the spheres of the same volume, after performing novel laboratory experiments on the gravitational settling of microplastic fibres.

The modelled deposition rates after coupling FES with respective nanoplastic emissions can be found in https://atmo-access.nilu.no/MPS_HLR1.py. To our knowledge, emission in the nanoplastic size range have not been published yet, thus the emissions used here refer to larger sizes (5–10 μm^36^). Previous work at the alpine station Sonnblick Observatory, Austria reported that the majority (~ 60%) f atmospheric PM_10_ microplastics is in the nanoparticle size range < 1 μm^18^. Therefore, we linearly scaled the emissions used here, so that the resulting modelled concentrations matched the observed deposition rates. Figure 4 depicts the results for the sites where the highest deposition rates were observed (sites 3, 8, 11, 13). Despite that the contribution of the ocean to the calculated FES was > 40%, the esulting modelled deposition rates that originate from sea from sea-spray are < 1%. At stes 3 and 8, France contributes to modelled deposition of nanoplastics by 46% and 5%, respctively, and Spain by 10% and 5%. At stes 11 and 13, which are characterised by the largest deposition rates (82 and 73 ng mL^− 1^), there is a relatively larger contribution to nanoplastic deposition from other countries closer to the Alps (e.g. Italy and Germany). For site 13, the contribution of Poland to the nanoplastics deposited in the Alps was also significant (12%), as ell as those of the UK and Ireland (16% and 5%, respctively).

## Outlook

In this study, snow from 14 high-altitude sites in the Alps near Mont Blanc was analysed by TD-PTR-MS for PE, PET, PP, PS, PVC and tire wear nanoparticles < 1 μm in size. Nanoplastics were detected at five of 14 sites, the majority being tire wear, PS and PE particles. The sampling by non-scientist mountaineers was shown to be feasible and compliant with quality assurance measures and we find that this sampling strategy pairs particularly well with TD-PTR-MS due to the small sample volume. Future adjustments could include a further level of blanks and defining a range of topographical structures to sample. With regard to TD-PTR-MS, LODs for PS and PET in the low ng range (similar to previous work) were determined, however, high-quality reference materials for more polymers are urgently needed for better LOD quantification and expansion of the plastics library. Lagrangian dispersion modelling shows that the origin of the nanoplastics are regions to the west of the Alps, with France, Spain and Switzerland having significant contributions. Dry and wet deposition both contribute to the observed deposition.

Overall, the citizen science approach is a promising way to gather data in remote and untravelled areas which are difficult for research teams to access without mountaineering or polar skills, and simultaneously raises awareness about plastic pollution among a wider audience. In principle, providing it is carefully supervised, crowd-sourcing of sample collection in this way enables multiple sites to be sampled within a short space of time. For this reason, the project is being expanded worldwide as the Global Atmospheric Plastics Survey 2024-25^40^. Combined with source region analysis, this would allow specific pollution-reducing measures to be implemented and monitored.

## Electronic supplementary material

Below is the link to the electronic supplementary material.

Supplementary Material 1

## Data Availability

All data needed to evaluate the conclusions in the paper, including the raw mass spectra data files and all stages of data analysis and processing, is available via DOI: 10.5281/zenodo.14180715. All FLEXPART results are openly accessible from https://atmo-access.nilu.no/MPS_HLR1.py.
