# Supplementary material for: Using a citizen science approach to assess nanoplastics pollution in remote high-altitude glaciers
Source: Sci Rep. 2025 Jan 13;15:1864. doi: 10.1038/s41598-024-84210-9 (PMC11729883; doi:10.1038/s41598-024-84210-9)
Supplement: Supplementary file 1 — Supplementary Material 1 [file 41598_2024_84210_MOESM1_ESM.docx]

**Using a citizen science approach to assess nanoplastics pollution in remote high-altitude glaciers**

Leonie Jurkschat^1,2^, Alasdair J. Gill^3^, Robin Milner^3^, Rupert Holzinger^4^, Nikolaos Evangeliou^5^, Sabine Eckhardt^5^ and Dušan Materić*^2^

^1^Faculty of Chemistry and Mineralogy, Leipzig University, Leipzig, 04103, Germany

^2^Department of Environmental Analytical Chemistry, Helmholtz Centre for Environmental Research (UFZ), Leipzig, 04318, Germany

^3^www.high-level-route.com

^4^Institute for Marine and Atmospheric Research (IMAU), Utrecht University, Utrecht, 3584, The Netherlands

^5^NILU, Department of Atmospheric and Climate Research (ATMOS), 2007, Kjeller, Norway

**Correspondence to*: Dušan Materić (dusan.materic@ufz.de)

Supplementary Information

# Experimental details of snow sampling

##
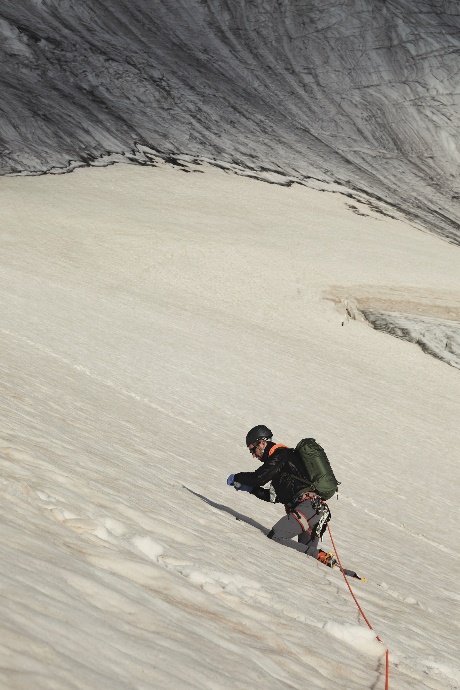
Standard Operating Procedure

Figure S1: Collection of surface snow from glacier. Photography: Zoe Salt (www.zoesalt.com).

Both team members are to remain roped up throughout the sampling process. The rope leader is primarily responsible for safety during the sampling process, whilst the second mountaineer (Nº2) must carry out sampling, recording and record-keeping (Figure S1 and S2).

1. When nearing a sample site, Nº2 prompts Leader to find a safe place to stop (avoiding obvious crevasses, rock fall or other hazards).
2. Leader decides when to stop the rope team, in collaboration with Nº2 (who will have a better view of the actual sampling site).
3. Leader remains constantly alert to possible crevasse falls, rock-fall or other hazards.
4. Nº2 extracts sampling kit (four vials plus HPLC water, notebook, camera, all in cotton bag) before moving to the actual sampling point, which shall be undisturbed snow, at least 5 metres from any obvious tracks.
5. Nº2 sets up camera on ice axe, with wide angle view of sampling site and starts video recording (optional, depending on conditions).
6. Nº2 dons gloves (new, nitrile), proceeds to take 3 samples from points forming a triangle with sides of between 1.5 and 3.0 metres.
7. Nº2 creates a “field blank” by part-filling a fourth vial with HPLC water from the bottle.
8. Nº2 calls out the reference codes of the vials, time, date, GPS position (from 2 devices) and atmospheric pressure (uncorrected for altitude, from 2 devices) and Leader notes down data.
9. Nº2 wraps the vials with aluminium foil and packs the used sample kit in their pack before making a final check that no equipment has been left, then returns to the normal track as defined by Leader.

##
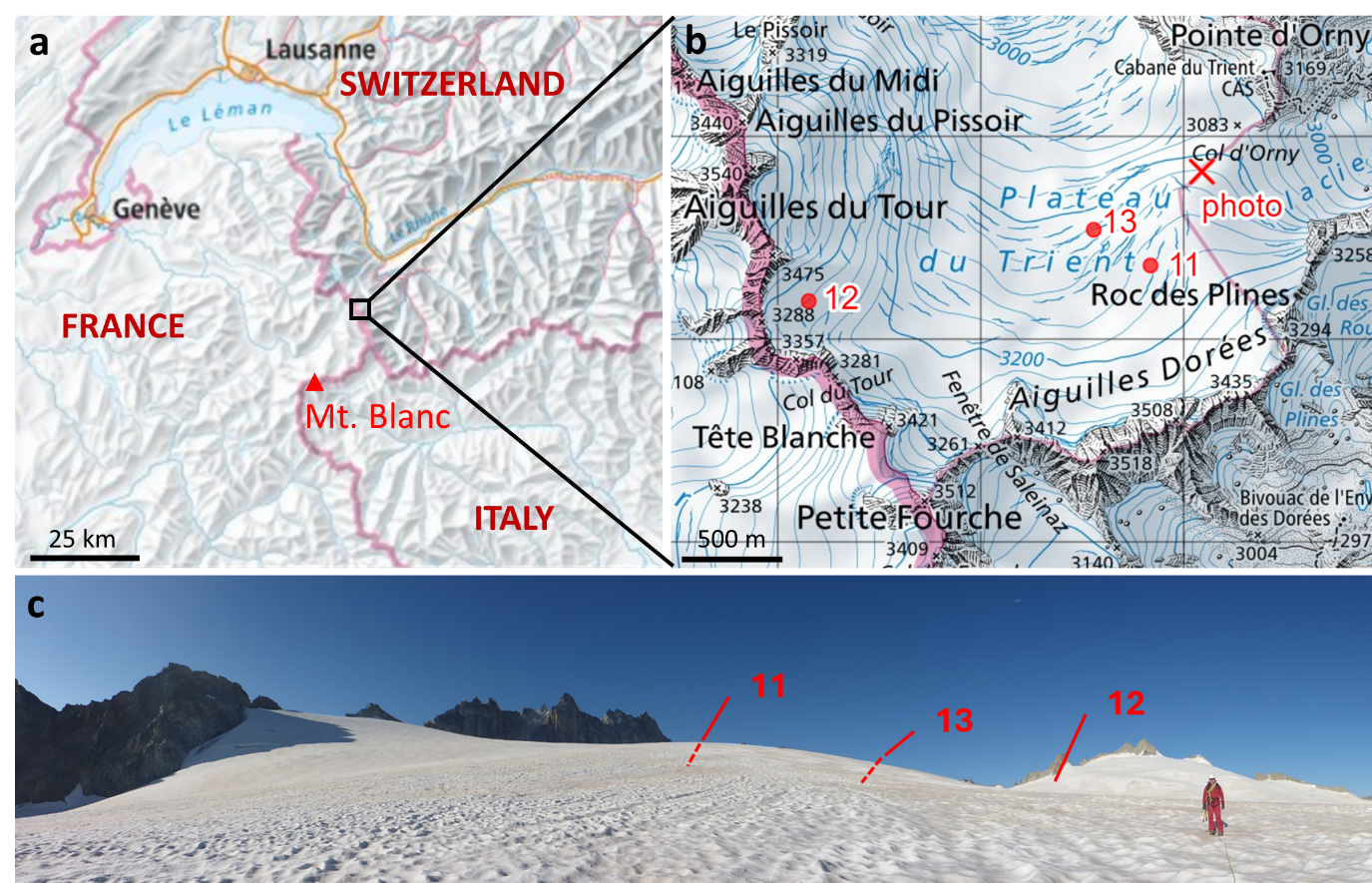
1.2 Location of sample sites 11–13

Figure S2: Location and topography at sites 11, 12 and 13. a. – b. Maps showing exact location of sample sites and point from which photo was taken. Created using www.map.geo.admin.ch. c. Panoramic photo indicating sample sites; site 11 and 13 are hidden behind near horizon.

# Plastic mass concentration calculated at each site

Table S1: Coordinates, altitude and calculated concentration of nanoplastics by polymer at each site. Value of each triplicate and each field blank (FB) given. Some measurements had to be excluded from analysis due to the instrument being out of ideal range of H_3_O^+^ ions, some vials were additionally measured in triplicate to test reproducibility; thus some sites have more or less than three data points and two field blanks had to be excluded from analysis.

|  |  |  | Calculated concentration of nanoplastics (ng mL^-1^) | | | | | |
| --- | --- | --- | --- | --- | --- | --- | --- | --- |
| Site | Coordinates | Altitude (m above sea level) | Tire | PVC | PS | PP | PET | PE |
| **1** | 45°51'7.5"N 06°56'31.7"E | 3408 | 0  0  0 | 0  0  0 | 0  0  0 | 0  0  0 | 0  0  0 | 0  0  0 |
| FB1 |  |  | 0 | 0 | 3.49 | 0 | 0 | 0 |
| **2** | 45°51'41.7"N 06°56'23.5"E | 3317 | 0  0 | 0  0 | 0  0 | 0  0 | 0  0 | 0  0 |
| **3** | 45°51'1.8"N 06°54'24.4"E | 3331 | 23.57  0  0  0  34.54 | 0  0  0  0  0 | 6.57  3.09  2.73  0  12.74 | 0  0  0  0  0 | 0  0  0  0  0 | 0  0  0  0  0 |
| FB3 |  |  | 0 | 0 | 0 | 0 | 0 | 0 |
| **4** | 45°50'59.5"N 06°53'28.5"E | 3552 | 0  0  0 | 0  0  0 | 0  0  0 | 0  0  0 | 0  0  0 | 0  0  0 |
| FB4 |  |  | 0 | 8.85 | 0 | 0 | 0 | 0 |
| **5** | 45°50'41.9"N 06°53'57.2"E | 3414 | 0  0  0 | 0  0  0 | 0  0  0 | 0  0  0 | 0  0  0 | 0  0  0 |
| FB5 |  |  | 0 | 0 | 0 | 0 | 0 | 0 |
| **6** | 45°50'48.0"N 06°54'17.3"E | 3374 | 0  0  0 | 0  0  0 | 0  0  0 | 0  0  0 | 0  0  0 | 0  0  0 |
| FB6 |  |  | 0 | 0 | 0 | 0 | 0 | 0 |
| **7** | 45°50'47.5"N 06°54'17.3"E | 3380 | 0  0  0 | 0  0  0 | 0  0  0 | 0  0  0 | 0  0  0 | 0  0  0 |
| FB7 |  |  | 0 | 0 | 7.44 | 0 | 0 | 0 |
| **8** | 45°50'58.9"N 06°55'39.9"E | 3405 | 0  0  0  0 | 0  0  0  0 | 0  0  0  8.40 | 0  0  0  0 | 0  0  0  29.43 | 0  0  0  0 |
| FB8 |  |  | 0 | 0 | 9.08 | 0 | 0 | 0 |
| **9** | 45°55'53.9"N 07°16'38.9"E | 3190 | 0  0  0 | 0  0  0 | 0  0  0 | 0  0  0 | 0  0  0 | 0  0  0 |
| FB9 |  |  | 0 | 0 | 9.07 | 0 | 0 | 0 |
| **10** | 45°55'52.9"N 07°16'33.3"E | 3124 | 0  0  0 | 0  0  0 | 0  0  7.64 | 0  0  0 | 0  0  0 | 0  0  0 |
| FB10 |  |  | 0 | 0 | 0 | 0 | 0 | 0 |
| **11** | 45°59'28.0"N 07°2'11.3"E | 3164 | 31.61  56.74  0 | 0  32.72  0 | 7.45  16.45  0 | 0  37.01  0 | 0  15.38  0 | 0  48.81  0 |
| **12** | 45°59'22.1"N 07°0'53.2"E | 3275 | 0  0 | 0  0 | 0  0 | 0  0 | 0  0 | 0  0 |
| FB12 |  |  | 0 | 0 | 8.40 | 0 | 0 | 0 |
| **13** | 45°59'33.7"N 07°1'58.2"E | 3121 | 79.82  29.69  16.49  30.69  21.56 | 0  0  0  16.32  0 | 26.04  5.74  2.65  6.08  4.95 | 41.97  0  0  0  0 | 25.38  0  0  0  0 | 56.83  0  0  0  0 |
| FB13 |  |  | 0 | 0 | 11.47 | 0 | 0 | 20.5 |
| **14** | 45°54'55.5"N 06°56'14.1"E | 1880 | 0 | 0 | 0 | 0 | 0 | 0 |
| FB14 |  |  | 0 | 0 | 0 | 0 | 0 | 0 |

# Spiking details

Table S2: Calculated masses of polystyrene (PS) of the four samples spiked with 300 ng. An average recovery/ionisation efficiency of 25 % was found. Reproducibility (σ) across the spiked the samples was 10.5 ng.

|  | Mass of PS calculated (ng) |
| --- | --- |
| spike field blank 6 | 81.7 |
| spike site 9 | 60.6 |
| spike site 10 | 82.8 |
| spike field blank 12 | 70.5 |
| **average (ng)** | **73.9** |
| **σ (ng)** | **10.5** |
| **average recovery/ionisation efficiency** | **24.6 %** |

# Single ion calibration and LOD calculation

Calibration measurements with standard materials (PS and PET) were made and the measured concentration of unique ions was used to define a calibration curve, as described in previous work [1]. The equivalent nanoplastic mass can thus be calculated from the 3σ limit from system blanks of this unique ion.


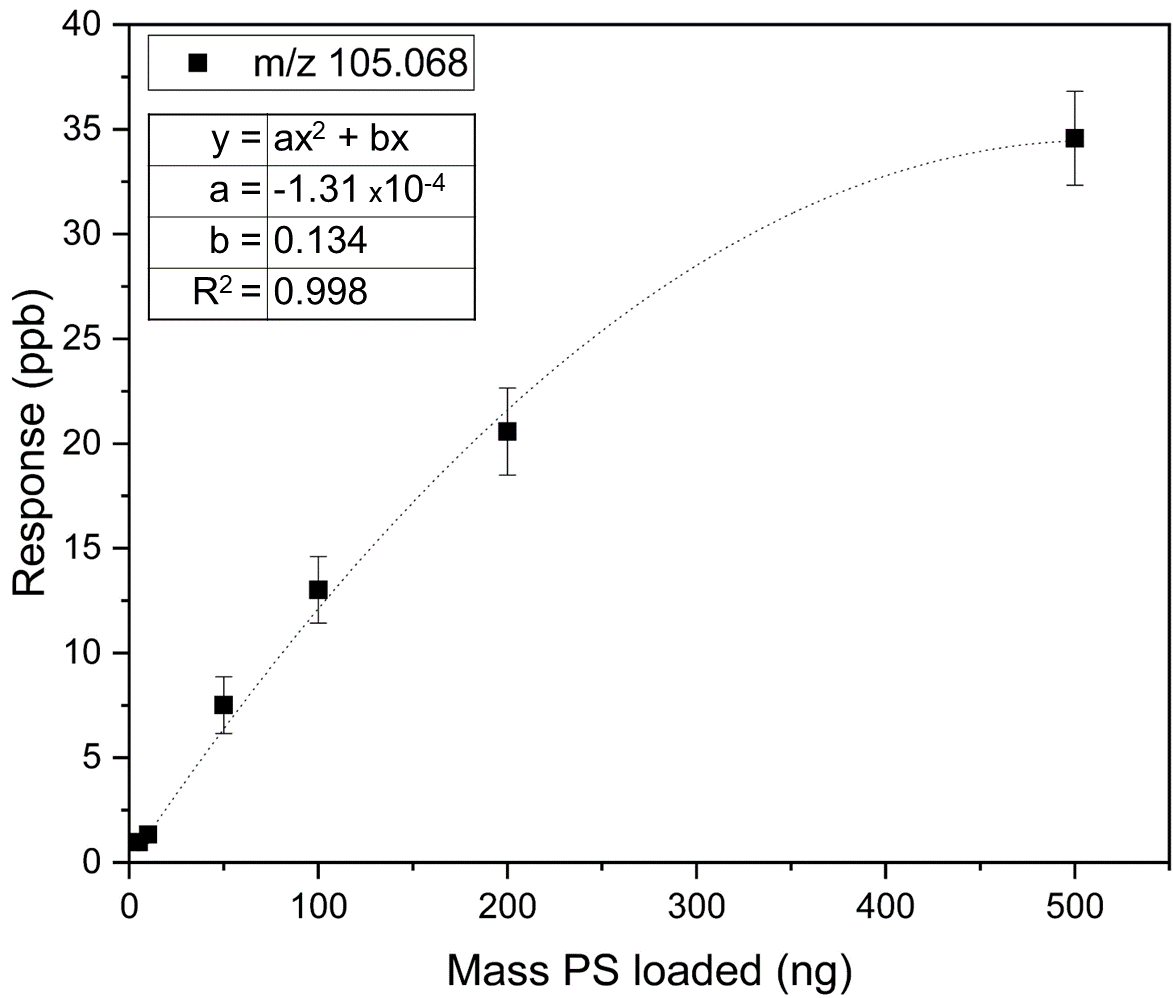
For PS (0.5 µm, analytical standard, Sigma-Aldrich), clean vials with 5, 10, 50, 100, 200 and 500 ng were prepared in triplicate and analysed using the same method described in Sec. 2.1. The same was done with a PET suspension that was available from another project [2], as no analytical standard is currently commercially available. The calibration curves are shown in Fig. S3 and S4 and the calculated LODs in Tab. S3.


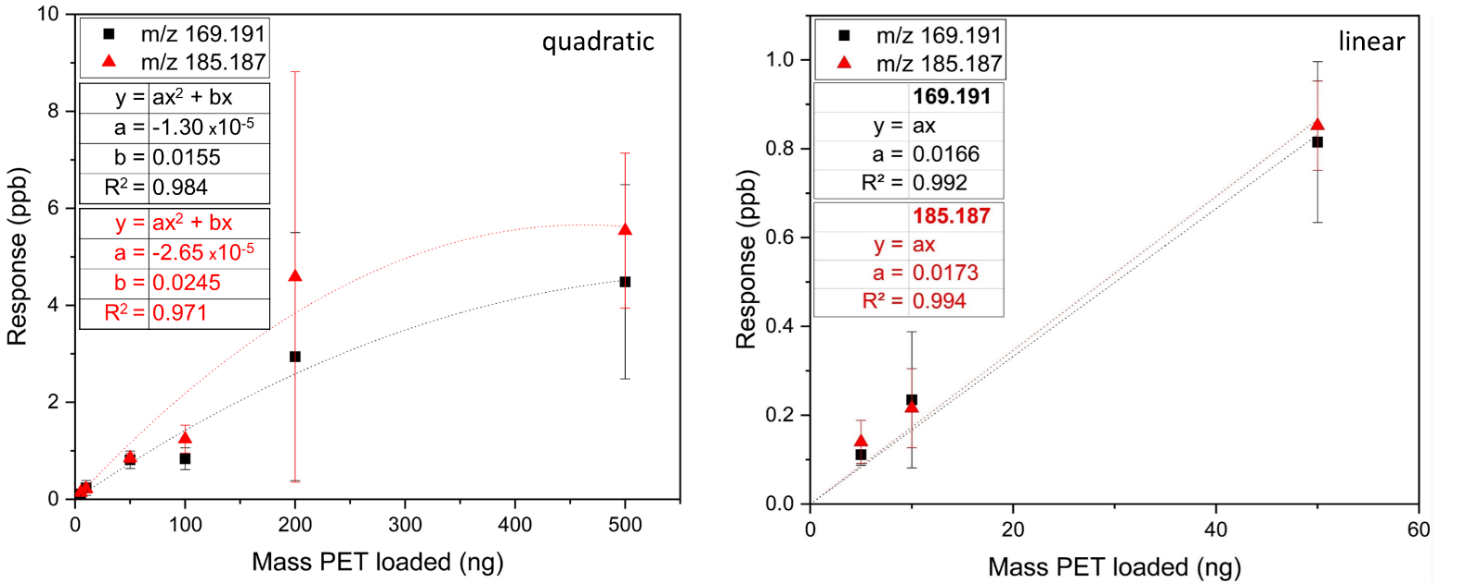
Figure S3: Calibration curve for polystyrene (PS), unique ion m/z 105.068.

Figure S4: Calibration curve for polyethylene terephthalate (PET), unique ions m/z 185.187 and 169.191. Range from 5 to 50 ng also calculated as linear curve.

Table S3: Calculated LODs for PS and PET using calibration curves (Fig. S3 and Fig. S4) of unique ions.

| Nanoplastic | Ion m/z | Calibration type | 3σ (ppb) | LOD (ng mL^-1^) |
| --- | --- | --- | --- | --- |
| PS | 105.068 | quadratic | 0.317 | 2.4 |
| PET | 185.187 | linear, 5–50 ng | 0.0166 | 1.2 |
|  | 169.191 | linear, 5–50 ng | 0.0108 | 1.1 |
|  | 185.187 | quadratic, 5–500 ng | 0.0166 | 0.7 |
|  | 169.191 | quadratic, 5–500 ng | 0.0108 | 0.7 |

# Sensitivity study


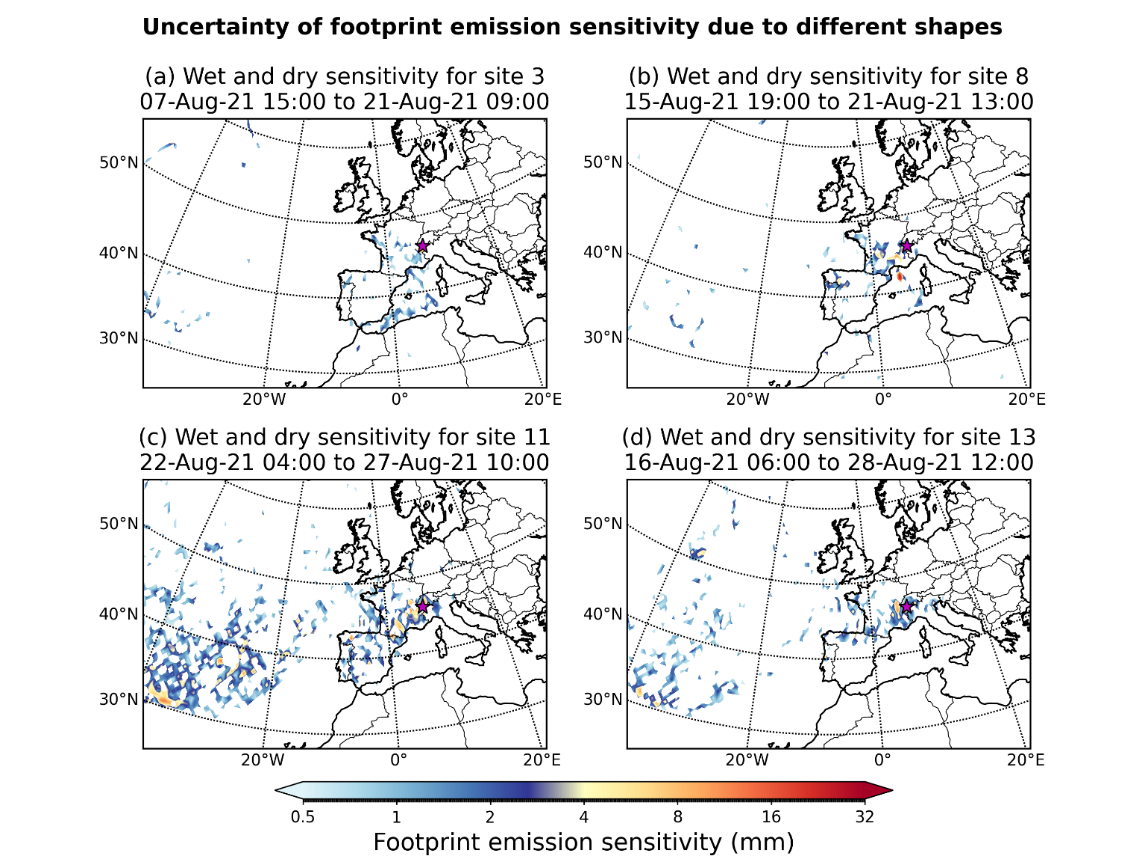
We performed a sensitivity study to calculate the uncertainty of transport due to the use of different shapes in the FLEXPART version 11 model. We simulated three different shapes, namely spheres with diameters 1 and 10 µm to match with the observations, fragments defined as cylinders with base diameters same as those of the spheres (1 and 10 µm) and length defined by an aspect ratio of 30, and fibres as cylinders of 1 and 10 µm diameter bases and aspect ratio of 100. The uncertainty was calculated as the standard deviation of the FES from the three different sensitivity experiments and is shown below.

Figure S5: FES uncertainty due to use of different shapes in FLEXPART version 11. We have simulated spherical particles of diameters same with the measured ones (1 and 10 µm), fragments as those defined in Methods Sec. 2.4 and fibres with 1 and 10 µm diameter base and an aspect ratio of 100. The uncertainty was calculated as the standard deviation of the FES inferred from the different simulations.

References

1. Materić, D. *et al.* Micro- and Nanoplastics in Alpine Snow: A New Method for Chemical Identification and (Semi)Quantification in the Nanogram Range. *Environmental science & technology* **54,** 2353–2359 (2020).

2. Villacorta, A. *et al.* A new source of representative secondary PET nanoplastics. Obtention, characterization, and hazard evaluation. *Journal of hazardous materials* **439,** 129593 (2022).
